# Mercury in bats from the northeastern United States

**DOI:** 10.1007/s10646-013-1150-1

**Published:** 2013-11-23

**Authors:** David E. Yates, Evan M. Adams, Sofia E. Angelo, David C. Evers, John Schmerfeld, Marianne S. Moore, Thomas H. Kunz, Timothy Divoll, Samuel T. Edmonds, Christopher Perkins, Robert Taylor, Nelson J. O’Driscoll

**Affiliations:** 1 BioDiversity Research Institute, 19 Flaggy Meadow Road, Gorham, ME 04038 USA; 2 U.S. Fish and Wildlife Service, Virginia Field Office, 6669 Short Lane, Gloucester, VA 23061 USA; 3 Center for Ecology and Conservation Biology, Boston University, 5 Cummington Street, Boston, MA 02215 USA; 4 University of Connecticut, 3107 Horse Barn Hill Rd. # U-4210, Storrs Mansfield, CT 06269 USA; 5 Trace Element Research Laboratory, Highway 60 VMA Building, College Station, TX 77843 USA; 6 K.C. Irving Environmental Science Centre, 32 University Ave, Wolfville, NS B4P 2R6 Canada

**Keywords:** Mercury, Hg, Methylmercury, MeHg, Bats, Northeast United States

## Abstract

This study examines mercury exposure in bats across the northeast U.S. from 2005 to 2009. We collected 1,481 fur and 681 blood samples from 8 states and analyzed them for total Hg. A subset (*n* = 20) are also analyzed for methylmercury (MeHg). Ten species of bats from the northeast U.S. are represented in this study of which two are protected by the Endangered Species Act (ESA 1973) and two other species are pending review. There are four objectives in this paper: (1) to examine correlates to differences in fur–Hg levels among all of the sampling sites, including age, sex, species, and presence of a Hg point source; (2) define the relationship between blood and fur–Hg levels and the factors that influence that relationship including age, sex, species, reproductive status, and energetic condition; (3) determine the relationships between total Hg and MeHg in five common eastern bat species; and (4) assess the distribution of Hg across bat populations in the northeast. We found total blood and fur mercury was eight times higher in bats captured near point sources compared to nonpoint sources. Blood–Hg and fur–Hg were well correlated with females on average accumulating two times more Hg in fur than males. On average fur MeHg accounted for 86 % (range 71–95 %) of the total Hg in bat fur. Considering that females had high Hg concentrations, beyond that of established levels of concern, suggests there could be negative implications for bat populations from high Hg exposure since Hg is readily transferred to pups via breast milk. Bats provide an integral part of the ecosystem and their protection is considered to be of high priority. More research is needed to determine if Hg is a stressor that is negatively impacting bat populations.

## Introduction

Mercury (Hg) in surface waters throughout the northeastern United States occur at relatively high concentrations and is released into the atmosphere in large part to due anthropogenic activities such as fossil fuel combustion, garbage incineration, gold mining, chlor-alkali, and textile manufacturing (Chen et al. 2005; Driscoll et al. 2007; Evers et al. 2007), while natural sources of atmospheric mercury provide a minor share (Schuster et al. 2002; Pirrone et al. 2010). Atmospheric deposition and waterborne point sources of Hg, in combination with environmental conditions that promote Hg-methylation and bioavailability, have led to the identification of several Hg hotspots in the Northeast (Chen et al. 2005; Evers et al. 2007). Forested regions may be particularly susceptible to high Hg levels owing to the filtering properties of the canopy and presence of wetlands that facilitate the bacterial transformation of Hg into methylmercury (MeHg)—a more biologically and ecologically relevant form (Driscoll et al. 2007). Numerous studies have reported on the distribution of Hg and MeHg in the United States (US), particularly in the northeastern region (Chasar et al. 2009; Evers and Clair 2005; Ward et al. 2010). Evers et al. (2007) identified five biological Hg hotspots in the northeastern United States (Maine, Massachusetts, Vermont, New Hampshire, New York) and one in southeastern Canada (Nova Scotia). In an effort to better understand the full-extent of the threat of Hg to wildlife in the Northeast, we evaluated bats as indicators of Hg bioavailability in terrestrial ecosystems.

Bats are excellent Hg bioindicators as: (1) many bat species are distributed across wide geographic ranges and while individuals of several species live in habitats that are relatively pristine, other individuals of the same species live near heavily industrialized areas or point sources of Hg emission; (2) most bat species are relatively long-lived (Brunet-Rossini 2004; Brunet-Rossini and Austed 2004; Wilkinson and South 2002; Kunz and Lumsden 2003) and so Hg may accumulate with age; (3) many bats of the Northeast are at high trophic levels making them susceptible to biomagnification (O’Shea and Johnson 2009; O’Shea et al. 2001); (4) bats may be exposed to higher Hg loads compared to other animals of similar size due to their high metabolic rate and food intake (Kunz 2004; Kunz et al. 1995; Kurta et al. 1989, Hickey et al. 2001; Wada et al. 2010). Mercury may also be of particular harm to bat populations as they have lower reproductive output compared with many other traditional study species, requiring adult survival for population stability (Barclay and Harder 2003; O’Shea and Johnson 2009). Additionally, bats are frequently subjected to multiple anthropogenic stressors and a number of species are endangered or threatened with extinction (Mickleburgh et al. 2002), or are experiencing rapid population loss.

While a large number of studies have explored Hg exposure to bats (Baron et al. 1999; Brooks and Ford 2005; Hickey et al. 2001; Miura et al. 1978; O’Shea et al. 2001; Petit and Altenbach 1973; Powell 1983; Wada et al. 2010; Walker et al. 2007), a knowledge gap remains with respect to spatial and temporal patterns of exposure and possible physiological effects. Blood and fur collected from bats at an anthropogenic Hg point source on South River, VA and a nearby reference site was analyzed by Wada et al. (2010). They found that mean concentrations of Hg in blood were significantly higher at the point source compared to a reference site. The mean value of Hg in bat fur [28.0 μg/g, fresh weight (fw)] was among the highest detected in wild mammals, and similar to other published mean values reported for bats captured at other point sources. The tri-colored bat, *Perimyotis subflavus*, which feeds over the North Fork of the Holston River in Virginia, a Hg point source, had significantly higher Hg concentrations in liver and muscle tissues compared to a control site (Powell 1983). Aquatic nymphs of flying insects from this river also had elevated Hg compared to areas upstream from the source (Powell 1983). Bat fur–Hg concentrations have been documented to exceed 30 μg/g for individuals sampled at areas with previously documented high Hg concentrations present in the environment (Miura et al. 1978; Grippo and Massa 2000).

This study examines Hg levels found in bats of the eastern United States. There are four objectives in this paper: (1) to examine correlates to differences in fur–Hg levels among all of the sampling sites, including age, sex, species, and presence of a Hg point source; (2) define the relationship between blood and fur–Hg levels and the factors that influence that relationship including age, sex, species, reproductive status, and energetic condition; (3) determine the relationships between total Hg and MeHg in five common eastern bat species; and (4) assess the distribution of Hg across bat populations in the northeast.

## Study area

**Fig. 1:**
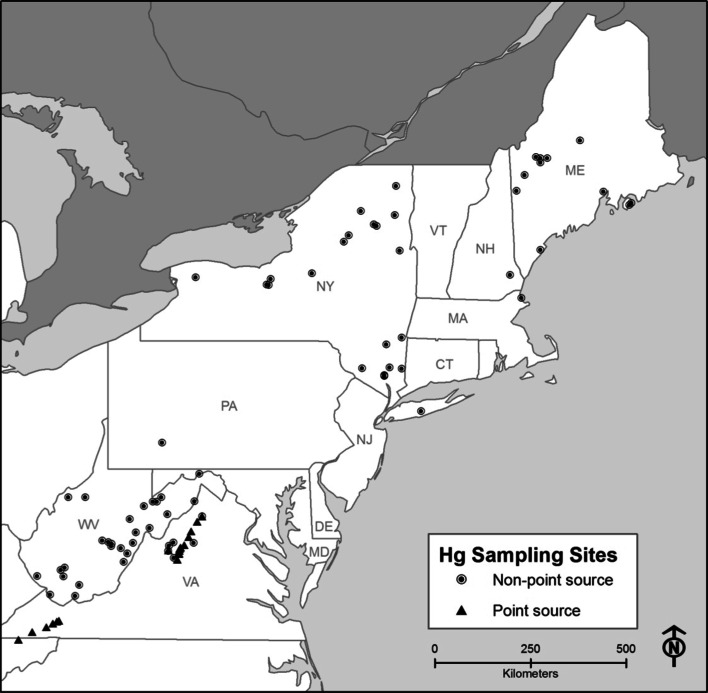
Sites sampled across the Northeast for Hg in fur and blood from bats (2006–2009)

Fur and blood samples were taken from bats in Maine, Maryland, Massachusetts, Virginia, New Hampshire, Pennsylvania, New York, and West Virginia at 89 sites between 2005 and 2009 (Fig. 1). Samples were collected at 20 sites with known anthropogenic Hg point sources upstream on two rivers in Virginia—the South River (textile, mercuric sulfate) and the North Fork of the Holston (chlor-alkali, mercuric chloride)—and at 69 sites with no recorded anthropogenic Hg inputs. The 20 sites in VA were defined as point-source contaminated areas using the definition given by the U.S. Environmental Protection Agency; as any stationary location or fixed facility from which pollutants are discharged or emitted or any single, identifiable discharge point of pollution, such as a pipe, ditch, or smokestack (US EPA 2010). Locations without a known point source were considered to be sites of atmospheric deposition. Fur samples were collected and analyzed for all of the seven states, while blood was only analyzed for sites in Maine, New York and Virginia.

### Capture, sample collection and handling

Bat capture, and fur and blood sampling were conducted between June and August in 2005–2009. Bats were captured using mistnets and harp traps (Constantine 1958) and also directly from roosts by hand. Beginning in 2007, USFWS white-nose syndrome decontaminant protocols were followed to prevent spread of *Pseudogymnoascus destructans*, the fungus believed to be responsible for high bat mortality rates in recent years (USFWS 2010). This protocol involved holding bats in single use disposable bags and disinfecting nets and equipment between trap sites (USFWS 2010).Fur samples were collected by trimming using stainless steel scissors that were cleaned and visually inspected between individuals to prevent cross contamination. Fur was stored in plastic zip bags (2 × 2 in.). Blood samples were collected by puncturing the acute ulnar or uropatagium vein with a sterile 27.5 gauge needle, and collecting the pooled blood in heparinized capillary tubes. Capillary tubes were sealed with Crito-caps, placed in labeled vacutainer tubes, set on ice, and stored at −20 °C until laboratory analysis. Individuals were then examined to determine age (Kunz and Anthony 1982) and sex, and measured [forearm length in millimeter (mm) and weight in grams (g)]. All bats were released onsite following collection.

### Mercury analysis

Total Hg concentrations were analyzed in sampled tissues. Fur–Hg was analyzed across all sites and for all years. Blood Hg was analyzed from bats sampled in 2007–2008. Laboratory analysis of THg in blood and fur (2005–2009) was conducted at the University of Connecticut (Center for Environmental Science and Engineering, Storrs, CT, USA) and the Biodiversity Research Institute (Gorham, ME. Quality control methods (including the use of external certified reference materials DOLT-4 and DORM-3) were used at both laboratories to ensure consistent analytical precision and accuracy. Recovery of total-Hg for all sample batches ranged from 90 to 110 %. Fur samples were analyzed for total Hg using a thermal decomposition technique with a direct Hg analyzer (DMA 80, Milestone Incorporated) using US EPA Method 7473 (Lesnik and Fordman 1998). Blood samples were analyzed for total mercury using cold vapor atomic fluorescence spectrophotometry (CVAFS) by EPA method 1631. Blood Hg concentrations are presented as wet weight (ww) and fur–Hg concentrations are presented as fw. Detection limits (DLs) for all batches were lower than 2.5 ng/g and all samples well exceeded this lower threshold.

Blood samples were digested with nitric and sulfuric acids at 95 °C for 10 min, oxidized with 0.02 N bromine monochloride, reduced with stannous chloride, bubbled onto a gold trap, and purged into a Brooks Rand Model III CVAFS for analysis. Sample mass for blood ranged from 0.006 to 0.02 g with an average detection limit of 0.01 ng/g, ranging from 0.004 to 0.4 μg/g, which were calculated according to standard EPA protocols. Standard quality assurance procedures were employed, including analysis of replicate samples [relative percent difference (RPD) = 17.0 %], method blanks (all below detection limit), spiked samples (percent recovery = 104.3 %), laboratory control samples (percent recovery = 106.0 %), quantitation limit samples, and standard reference material recovery (DOLT-3 = 95.0 % and DORM-2 = 102.5 %, National Research Council, Canada; SRM 966 = 100.5 %, National Institute of Standards and Technology) the average RPD was 9.7 %. Instrument response was evaluated initially, every 20 samples, including quality control samples, and at the end of an analytical run using calibration verification standard and blank.

Methylmercury in fur was analyzed in subset of fur samples collected from one of the non-point source sites in VA to provide an approximation of the percentage of MeHg of the total-Hg in fur. Analysis for Hg species in fur was completed at Acadia University, Nova Scotia following digestion in 25 % KOH/MeOH, similar to methods described by Liang et al. (1994), Edmonds et al. (2010). Quality control included method replicates (mean COV = 5 %, 3 %; *n* = 2, [MeHg; THg]), analytical triplicate (mean COV = 15 %, 14 %; *n* = 1), method blanks [DLs = 0.23 pg MeHg; 0.46 pg Hg(II)], and certified reference material (DOLT-4, National Research Council, Canada, mean recovery = 101 % MeHg, 104 % THg, *n* = 3).

### Statistical analysis

For our objectives we chose to use linear mixed models to test specific hypotheses about the factors that influence fur–Hg levels in bats across multiple sites and species, and the relationship between blood and fur–Hg levels at the few sites where both types of tissues were collected. For our analysis of Hg in fur across all sites, we tested the hypothesis that fur–Hg levels changed due to species, bat age, bat sex, and whether the site was in close proximity to a known Hg point source as all of these variables were collected in all the data sets. In this case, it made sense to make site and year random variables because sampling was erratic across those two factors and we know there were other differences among sites that altered Hg availability that were not quantified (e.g. habitat, water quality, soil composition, etc.). The model we tested included all the variables listed as base effects and also included interactions among site type and age, and site type and sex. We tested the interactions between sex, age and whether the site was a point source. We tested all possible interactions and used this model to evaluate our hypotheses. Instead of testing multiple models, we included variables into the model based on expert opinion then reported that model without removing individual terms with *p* values greater 0.05. While this method can lead to a model that has less predictive utility due to higher model error, this method is reasonable for testing specific hypotheses. The model presented here cannot be applied to predict Hg levels in bats outside of our study area.

For our second analysis we explored the relationship between blood–Hg and fur–Hg levels and the factors that influenced those relationships. We initially created a simple linear regression to look at overall correlation between blood and fur–Hg levels in adult and juvenile bats, but we also wanted to test how a variety of factors influenced the blood/fur relationship in a controlled modeling environmental. Specifically, we tested whether fur is an appropriate indicator of blood Hg for each captured species, both sexes, adults and juveniles, throughout the different reproductive stages of the breeding season (pregnant, lactating, etc.) and bats of a variety of sizes. To do this, we constructed another general linear mixed model with the primary objective being to determine the relationship between blood and fur–Hg levels in bats and how these factors might interact with this relationship. We used a subset of our total bat Hg database (those that have both blood and fur samples analyzed) so we could use a broader set of possible variables in this analyses without sacrificing sampling size. Even though there were only six sites used in this analysis, we included site as a random variable here to control for any unquantified site-specific effects. Like the first model, we developed a series of testable hypotheses a priori and included all in the final model regardless of significance where we can evaluate each relationship controlling for all other tested possibilities.

All Hg data was transformed using a natural logarithm to meet the normality requirements of the tests. All statistical analyses were performed using JMP 9.3 statistical program (SAS Institute 1985).

## Results

A total of 2,128 tissue samples were collected and analyzed for Hg, including 1,481 fur samples and 681 blood samples from 1,447 bats. Fur was collected from ten bat species and blood was collected from seven bat species. The mean fur total Hg concentration from the anthropogenic point source sites was 52.46 μg/g (*n* = 600, range 0.38–707.64 μg/g, SD = 89.03), while at atmospheric de position sites the mean was 6.44 μg/g (*n* = 881, range 0.07–120.31 μg/g, SD = 8.71). The blood total-Hg mean from point source sites was 0.47 μg/g (*n* = 393, range 0.002–3.76 μg/g, SD = 0.75) and the mean from the non-point sources was 0.05 μg/g (*n* = 288, range 0.002–0.55 μg/g, SD = 0.05). The highest Hg means in fur and blood were detected in tri-colored (*Perimyotis subflavus*), little brown (*Myotis lucifugus*) and northern long-eared (*Myotis septentrionalis*) (Table 1).

**Table 1:** Total -Hg concentrations in fur (μg/g, fw) and blood (μg/g, ww) by species in the Northeast U.S.

	Total blood Hg						Total fur–Hg					
Species	*n*	Mean	25 % quantile	Median	75 % quantile	Max	*n*	Mean	25 % quantile	Median	75 % quantile	Max
Tri-colored	75	40.77	4.92	15.30	52.21	255.00	29	0.74	0.12	0.42	1.09	2.75
Little brown	851	29.22	2.49	5.39	15.69	707.64	410	0.28	0.02	0.04	0.18	3.76
Northern long-eared	220	26.89	3.40	7.37	16.82	480.00	82	0.60	0.06	0.12	0.81	3.70
Gray	7	18.61	2.70	5.37	24.80	84.50	7	0.12	0.01	0.02	0.24	0.46
Big brown	203	16.64	4.59	9.59	18.10	200.00	127	0.10	0.04	0.06	0.11	0.89
Indiana	12	10.58	6.17	10.35	16.04	18.30	–	–	–	–	–	–
Eastern small-footed	7	12.88	7.54	15.70	16.50	18.83	–	–	–	–	–	–
Silver-haired	6	7.96	5.15	7.89	10.17	14.23	–	–	–	–	–	–
Red	54	4.03	1.29	2.73	5.22	25.54	20	0.05	0.01	0.03	0.06	0.22
Hoary	12	1.33	0.66	1.34	1.71	3.61	6	0.02	0.01	0.01	0.03	0.03

**Fig. 2:**
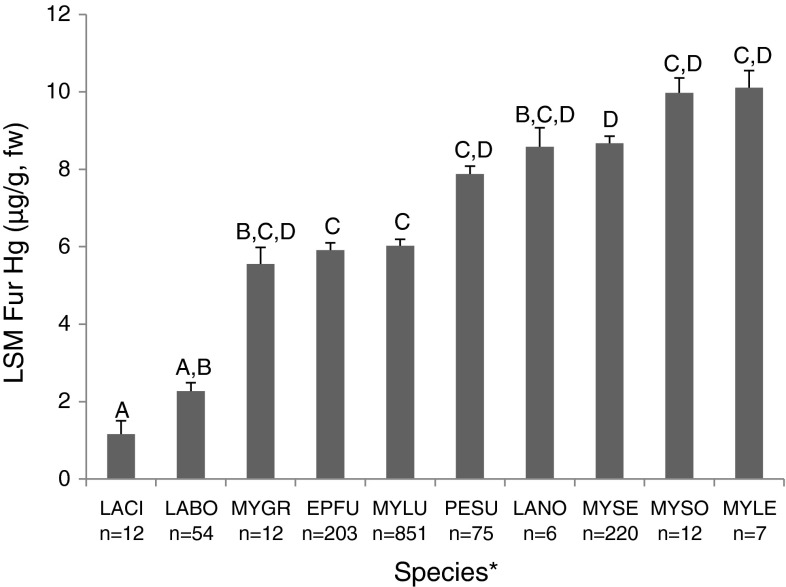
Least square means of Hg (μg/g, w) in fur from bats separated by species with standard *error bars*. *Letters* within the mean *bars* represent the results of post hoc Tukey HSD test; *bars* *that share common letters* do not differ significantly. *LACI hoary, *LABO* red, *MYGR* gray, *EPFU* big brown, *MYLU* little brown, *PESU* tri-colored, *LANO* silver-haired, *MYSE* northern long-eared, *MYSO* Indiana, *MYLE* eastern small-footed

**Fig. 3:**
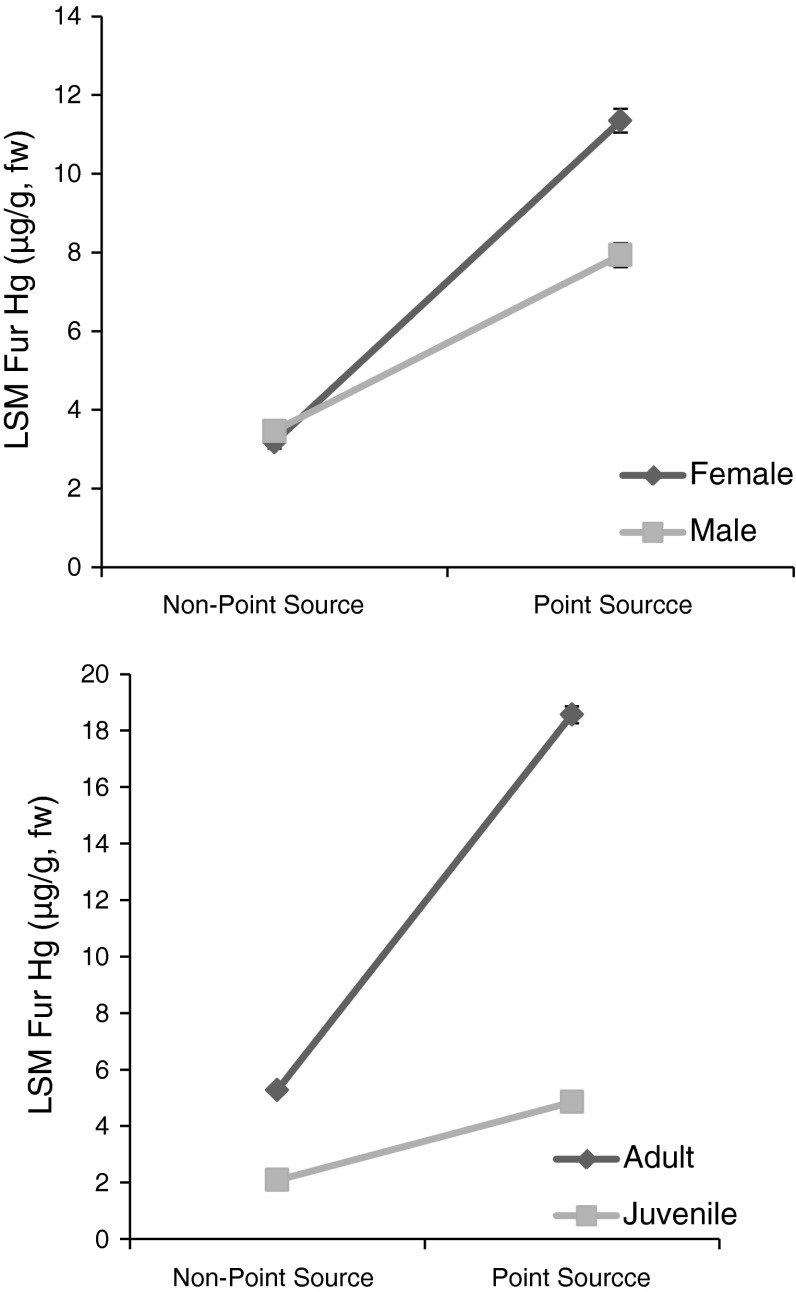
Least square means of Hg (μg/g, w) in fur showing sex and age at point source and non-point source sites with standard *error bars*

Our first model showed good overall fit and suggests that species, age, sex and site contamination were all important to predicting fur–Hg levels (adj. r^2^ = 0.494). Controlling for site and year we found that there were significant differences among species fur–Hg levels (F(9,1155) = 11.74, *p* < 0.001). Myotis species—like eastern small-footed bat (*Myotis leibii*), Indiana bat (*Myotis sodalis*) and little-brown bat—tended to have the highest levels while migratory tree bats—like red (*Lasiurus borealis*), hoary (*Lasiurus cinereus*)and silver-haired bats (*Lasionycteris noctivagans*)-were the lowest (Fig. 2; Table 2). Site type, age and sex were tested together in all possible interactions and all were significant (Table 2). Adult females at contaminated sites had the highest average fur–Hg levels at 23.5 ± 1.4 SE μg/g while adult males at such sites only averaged 13.4 ± 1.4 SE μg/g. Juvenile males and females showed small differences at these sites also, females averaged 4.8 ± 1.4 μg/g SE males averaged 6.4 ± 1.4 μg/g SE. At uncontaminated sites, adults and juveniles showed differences (e.g. 5.2 ± 1.7 SE μg/g in adult females compared to 2.0 ± 1.2 SE μg/g in juvenile females). Year accounted for 2 % of the total variance whereas site accounted for 17 %, suggesting differences among sites that we did not account for with our fixed effects. These data suggest that point source sites have a significant effect on Hg levels, in particular for adult Myotis females (though adult males and other species could also have high levels) (Fig. 3).

**Table 2:** Modeled effect size of fur–Hg levels across all factors in the top model

Modeling term	Estimate	S.E.	Prob > *t*
Intercept	1.75	0.19	<0.0001
Sex [female]	0.01	0.04	0.89
Age [adult]	0.52	0.04	<0.0001
Point source/non-point source [point source]	0.54	0.15	0.00
Tri-colored	0.39	0.15	0.01
Big brown	0.04	0.12	0.73
Red	−0.89	0.16	<0.0001
Hoary	−1.55	0.28	<0.0001
Silver-haired	0.44	0.42	0.30
Gray	−0.06	0.37	0.86
Eastern small-footed	0.58	0.38	0.12
Little brown	0.05	0.10	0.62
Northern long-eared	0.44	0.11	0.00
Point source/non-point source × sex	0.06	0.04	0.12
Point source/non-point source × Age	0.07	0.04	0.09
Sex × age	0.12	0.04	0.00
Sex × age × contaminated/uncontaminated	0.09	0.04	0.01

Categorical variables are relative to the unmentioned group (e.g., males for sex, juveniles for age, non-point source for point source presence and *M. sodalis* for species)

**Fig. 4:**
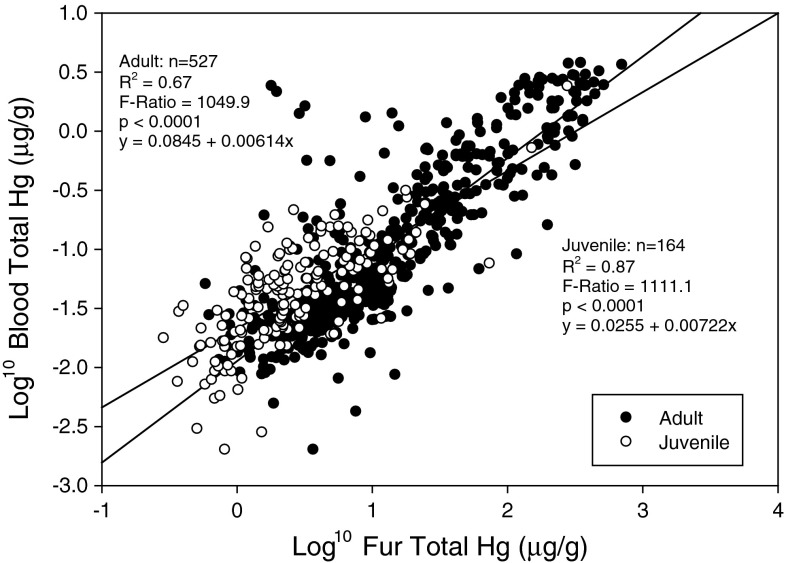
Correlations between concentration of Hg in blood and fur from juvenile and adult bats

Without covariates, blood–Hg and fur–Hg levels were well-correlated overall (adult r^2^ = 0.67, *p* < 0.0001, juvenile r^2^ = 0.87, *p* < 0.0001; Fig. 4). Our model describing the blood–Hg/fur–Hg relationship fit well and showed significant relationships among all of the base variables we selected and good overall fit (adj. r^2^ = 0.785). Blood Hg was highly positively correlated with fur–Hg overall and was the most important single variable. With a beta value of 0.597 ± 0.132, fur is a good, positive predictor of blood Hg overall (F(1,658) = 20.5, *p* < 0.001), however our model has many possible interactions with this term and cannot be considered alone (Table 3). Species was highly important to the blood/fur relationship (F(6,658) = 2.35, *p* = 0.03). Tri-colored and red bats had lower blood levels than would be predicted by the average blood/fur relationship and gray bats had higher blood levels than predicted. Contaminated sites had much higher blood levels than would be predicted by average fur levels (F(1,644) = 25.8. *p* < 0.001). Blood Hg levels changed with reproductive stage (F(3,660) = 1.98, *p* = 0.117; though see Table 4 for individual parameter estimates), non-reproductive bats had higher levels than all other stages, however there is also some evidence that the blood/fur relationship varies also varies with reproductive stage (F(3,658) = 2.16, *p* = 0.09). Non-reproductive bats had a lower correlation between blood/fur levels. Sex, age and body mass had no effect on the blood/fur relationship.

**Table 3:** Modeled effect size of fur–Hg levels across all factors in the top model

Modeling term	F-ratio	DF	Prob > *F*
Contaminated/uncontaminated	2.46	1	0.17
Species	7.01	6	<0.0001
Sex	0.58	1	0.45
Age	0.04	1	0.84
Body mass	2.23	1	0.14
Forearm length	0.10	1	0.75
Reproductive status	1.97	3	0.12
Fur mercury level	20.52	1	<0.0001
Fur mercury × contaminated/uncontaminated	25.82	1	<0.0001
Fur mercury × species	2.35	6	0.03
Fur mercury × sex	0.20	1	0.66
Fur mercury × age	0.11	1	0.74
Fur mercury × body mass	1.29	1	0.26
Fur mercury × reproductive status	2.16	3	0.09

Categorical variables are relative to the unmentioned group (e.g. males for sex, juveniles for age, non-point source for point source presence and *M. sodalis* for species)

**Table 4:** Modeled effect size of fur–Hg levels across all factors in the top model

Modeling term	Estimate	S.E.	Prob > *t*
Intercept	−0.92	0.99	<0.0001
Contaminated/uncontaminated [contaminated]	−0.24	0.15	0.17
Tri-colored	1.89	0.39	<0.0001
Big brown	−0.23	0.32	0.48
Red	−0.14	0.23	0.54
Hoary	−1.03	0.64	0.11
Gray	−1.00	0.42	0.02
Little brown	0.03	0.27	0.90
Sex [female]	0.06	0.08	0.45
Age [adult]	−0.02	0.09	0.84
Body mass	0.07	0.05	0.14
Forearm length	0.01	0.02	0.75
Reproductive status [lactating]	−0.11	0.12	0.37
Reproductive status [not reproductive]	0.31	0.14	0.03
Reproductive status [pregnant]	−0.13	0.24	0.58
Fur mercury level	0.60	0.13	<0.0001
Fur mercury level × contaminated/uncontaminated [contaminated]	0.16	0.03	<0.0001
Fur mercury level × tri-colored	−0.30	0.17	0.08
Fur mercury level × big brown	−0.04	0.17	0.80
Fur mercury level × red	−0.25	0.18	0.16
Fur mercury level × hoary	0.36	0.69	0.60
Fur mercury level × gray	0.26	0.20	0.20
Fur mercury level × little brown	0.00	0.15	0.99
Fur mercury level × sex [female]	0.01	0.03	0.66
Fur mercury level × age [adult]	0.01	0.04	0.74
Fur mercury level × body mass	−0.02	0.02	0.26
Fur mercury level × reproductive status [lactating]	0.05	0.04	0.22
Fur mercury level × reproductive status [not reproductive]	−0.08	0.05	0.08
Fur mercury level × reproductive status [pregnant]	0.07	0.09	0.42

Categorical variables are relative to the unmentioned group (e.g. males for sex, juveniles for age, non-point source for point source presence, post-lactating for reproductive status and *M. sodalis* for species)

### Percent MeHg in Fur

**Fig. 5:**
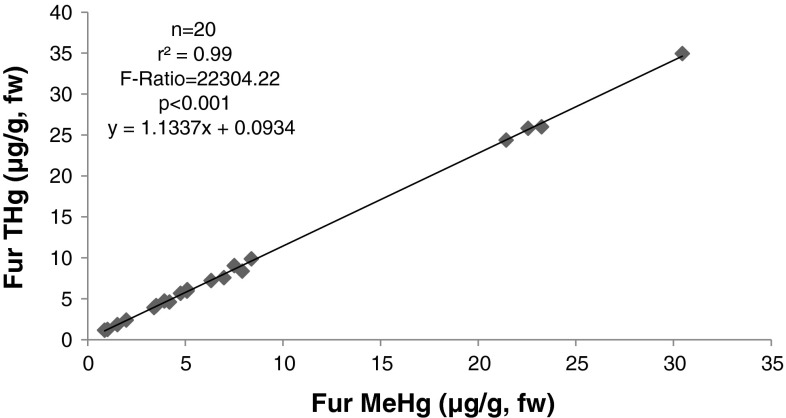
Correlation between total Hg and MeHg in adult bats

MeHg was measured in fur from 20 bats captured at a non-point source site in Virginia to estimate the percent MeHg of total Hg. MeHg ranged from 71 to 95 % fur–Hg (mean 86 %). Five species were sampled, big brown (*Eptesicus fuscus*), red, little brown, northern long-eared and tri-colored, but sample sizes were too small to make a comparison among species (Table 5; Fig. 5). The trend of higher total Hg and MeHg exhibited a strong positive correlation and showed that when THg increased MeHg increased at a similar rate almost a 1:1 ratio (r^2^ = 99, F = 22304.22, *p* < 0.001).

**Table 5:** Mean (±SD) total Hg (μg/g, fw), MeHg (μg/g, fw) and MeHg percentages from bat fur separated by species

Species	*N*	Mean total Hg	Mean MeHg	Mean MeHg (%)
Big brown	5	9.89 (±9.44)	8.61 (±8.54)	85 (±2 %)
Red	3	3.76 (±1.21)	3.15 (±1.03)	84 (±1 %)
Little brown	4	18.89 (±13.17)	16.43 (±11.60)	86 (±2 %)
Northern long-eared	6	7.11 (±9.35)	6.19 (±8.20)	85 (±6 %)
Tri-colored	2	7.97 (±0.56)	7.45 (±0.66)	93 (±2 %)

## Discussion

Bats captured at point sources in the northeastern US had significantly higher mean Hg concentrations in fur and blood compared to those from non-point source Hg sites. When all species and age classes were combined, the mean concentrations of Hg in fur from the combined anthropogenic point sources (52.7 μg/g, fw) were almost eight times higher than from non-point sources (6.7 μg/g, fw). The mean concentration of Hg in blood found at the combined point sources (0.47 μg/g, ww) was ten times higher than the mean of non-point sources (0.047 μg/g, ww). Other researchers have reported the concentrations of Hg in bats (Baron et al. 1999; Brooks and Ford 2005; Hickey et al. 2001; Miura et al. 1978; O’Shea et al. 2001; Petit and Altenbach 1973; Powell 1983; Wada et al. 2010; Walker et al. 2007), however, the present study provides the largest dataset on Hg concentrations in fur and blood from bats to date.

Blood total Hg concentrations generally represents recent dietary uptake of MeHg; fur–Hg concentrations indicates Hg at the time of fur growth in which the Hg is remobilized by muscle and organs and sequestered in growing fur (Evers et al. 2005; Mierle et al. 2000; Yates et al. 2005). Concentrations of Hg in fur is an expression of chronic exposure (Mierle et al. 2000), the total Hg concentrations in the fur of juveniles were lower than in adults which indicates accumulation over time. This could explain the significant difference in Hg concentrations in the fur of adult and juvenile bats from the point sources. When comparing point source and non-point source sites, only juvenile little browns differed significantly in mean concentrations of Hg in fur and blood. Adults of all species differed significantly between point and non-point source sites except for red (fur and blood Hg), hoary (fur–Hg) and tri-colored bats (blood Hg). Body weight and Hg concentrations were negatively correlated in bats from point source sites, which suggest that bats maybe able to depurate Hg more efficiently through excreting it in the keratin in their fur (Wada et al. 2010). Conversely, if larger bats have lower fur–Hg it could be due in part to a dilution effect where the larger body diluting the contaminant or that the bat is feeding at a lower trophic position on the insects that are not accumulating as much Hg.

Bats accumulate the majority of Hg through their diet of emergent insects, using both aerial and gleaning foraging techniques over river surfaces and floodplain edges (Baron et al. 1999). The difference in means for fur and blood–Hg between species within a site is likely due to differing feeding strategies and prey choice. A 2003 dietary study of bats showed that common prey items of big brown, little brown and tri-colored bats had varied diets, and includes Coleoptera, Hemiptera, Lepidoptera, Homoptera, Diptera, Hymenoptera and Trichoptera. Diets for these bats did not differ from diets in other regions of the United States (Carter et al. 2003). In New Hampshire, the diet of little brown bats includes insect orders Diptera, Hemiptera, Trichoptera, Lepidoptera, Coleoptera, and Arachnida (Anthony and Kunz 1977). In Maryland and Pennsylvania, big brown bat has a varied diet, but predominately feeds on Coleoptera (Agosta and Morton, 2003). In Ontario, red bats also have a varied diet, consuming over 127 different species of insects, and represent eight orders (Clare et al. 2009). Hoary bats consume prey similar to big brown, little brown and tri-colored while migrating through New Mexico, with the exception of Trichoptera (Valdez and Cryan 2009). Carter et al. (2003) found that northern long-eared and red bats also prey upon Coleoptera and Lepidoptera. Other studies found that the main prey of northern long-eared bat was Coleoptera and Lepidoptera, but overall had a varied diet, including arachnids (Brack and Whitaker 2001; Whitaker and Hamilton 1998). Similar to little brown and tri-colored, Indiana bats have a varied diet in Missouri and Indiana including: Lepidoptera, Coleoptera, Trichoptera, and Diptera. Spiders and Lepidoptera larva were also found in the stomachs of this species from a cave in Indiana (Brack and Whitaker 2001). Arachnids had higher Hg concentrations than other terrestrial invertebrates collected at the South River, Virginia (Cristol et al. 2008) and could explain why *Myotis* sp. had some of the highest levels of Hg in this study. Red and hoary bats consistently had the lowest mean concentrations of Hg in fur and blood when compared to other species included in our study. In addition to prey preferences and migratory behavior may also result in lower concentrations of Hg in blood and fur in species of Lasiurus. Given that species of Lasiurus examined in the present study are highly territorial, they are not likely to forage over the same point source rivers for extended periods of time and some of the bats captured may have been new to the area where they were sampled, explaining their lower fur and blood Hg concentrations.

Differences between male and female bats were detected in mean concentration of total Hg in fur and blood from both point source and non-point source sites. We hypothesize that female bats exhibited higher Hg concentrations than males at point source site due to decreased foraging distances (thus closer proximity to the point source) during pup rearing. This can also partially explain why female bats at point source sites had significantly higher Hg than female bats at non-point source sites. Since females depurate Hg through birth and milk production it was thought that they may have lower total Hg burdens but this was not the case in this study. This finding could have larger implications since reproducing females are more susceptible to accumulating Hg. Mercury is readily transferred across the placenta, and concentrates selectively in the fetal brain. Yang et al. (1972) found Hg concentrations in the fetal brain of rodents fed MeHg were twice as high as in the maternal brain. Reproductive effects of MeHg in mammals range from developmental alterations in the fetus, which produce behavioral or physical deficits after birth, to death (Chang et al. 1974; Chang and Annau 1984; Eccles and Annau 1987; Khera 1979; Wren et al. 1987). These effects could lead to a decrease in bat reproductive success, especially considering the reported high female Hg levels.

Procella et al. (2004) found that MeHg ratios were highest in the fur of raccoons (99 % of THg) compared to blood, brain, heart, kidney, liver and muscle samples. In otter and mink, percentages in fur were 79 and 65 %, respectively, and were not the highest in fur when compared to brain, kidney and liver sample (Evans et al. 2000). We found that MeHg in the fur of bats sampled fell within a similar range of 71–95 %. Concentrations of MeHg in human hair and bat fur are highly correlated with concentrations in blood (Clarkson and Magos 2006).

Based on Hg exposure profiles for bats in 2006–09 in the present study, there is compelling evidence of Hg at point source and some non-point sources having the potential to have an adverse affect on insectivorous bats. Dong-Ha Nam et al. (2012) found bats had Hg-associated neurochemical changes with range of 10–40 μg total Hg/g, fw, threshold in fur. Sixty-nine percent of the Hg levels in fur of adult bats that we analyzed from point sources exceed 10 μg/g, fw, whereas only 21 % of the adult bats have Hg levels in fur from the non-point sources are above the 10 μg/g, fw threshold. Other small mammals including mice have similar neurochemical affects from being exposed to Hg. Burton et al. (1977) found that wild mice, eating brine flies from the Great Salt Lake, had Hg concentrations over 7.8 μg/g, fw in fur exhibited behavioral deviations and had a decrease in ambulatory activity when compared to a control group. Burton et al. (1977) also found that mice with Hg concentrations in fur of 10.8 μg/g, fw showed decreased stress tolerance and decreased swimming ability. Eighty-one percent and 32 % of the adult bats from the point and non-point sources had Hg concentration that exceeded the effect level of 7.8 μg/g, fw, respectively.

Bats are increasingly of high conservation concern owing to the impacts of various anthropogenic influences (Jones et al. 2009). Mercury is an anthropogenic stressor on bats that may be compounded by other stressors such as white-nose syndrome, a syndrome that has been causing mass mortality among hibernating bats throughout the Northeast and Mid-Atlantic states (Frick et al. 2010). Thus, future investigations are important to determine spatially and temporally explicit effects from Hg with high resolution of reproductive success, survival, and physiological responses to emerging pathogens and other stressors or contaminants are of considerable importance to bat conservation. More studies examining the effects of Hg on bats are needed to quantify if bats are being affected by these elevated Hg concentrations. Two of the bats sampled [Indiana and gray (*Myotis grisescens*)] in the study are already protected by the Endangered Species Act (ESA 1973) and two more are pending (eastern small-footed, northern long-eared) with possibly a third under consideration in the near future (little brown). All had elevated Hg concentrations which may be of concern. Bats provide important ecosystem services and are a keystone species (Boyles et al. 2011; Kunz et al. 2011), and their protection must be made a high priority.
